# The Game of Skittles on the Northern Route of the Camino de Santiago

**DOI:** 10.3389/fpsyg.2020.588223

**Published:** 2020-12-18

**Authors:** José E. Rodríguez-Fernández, Mar Lorenzo-Moledo, Jesús García-Álvarez, Gabriela Míguez-Salina

**Affiliations:** ^1^Department of Applied Didactics, University of Santiago de Compostela, Santiago de Compostela, Spain; ^2^Department of Pedagogy and Didactic, University of Santiago de Compostela, Santiago de Compostela, Spain

**Keywords:** skittles, traditional sports games, intangible cultural heritage, Camino de Santiago, Northern Camino, pilgrimage, heritage

## Abstract

The main purpose of this study was to analyze the presence and current situation of the game of skittles throughout the northern route of the Camino de Santiago. Thus, we considered its current practice, modalities, where it is played, and its different manifestations as an informal and formal game (sport), comparing it with other traditional games on this pilgrimage route. To do this, a mixed qualitative-quantitative study was designed with 89 participants (municipal professionals, politicians, players, club managers, and teachers), constituting an informant for each municipality through which the Northern Way passes. An *ad hoc* questionnaire was used for the data collection, which was processed through content analysis by expert judges (qualitative section) and by using the IBM-SPSS statistical package (version 25). The results of the study show notable skittles activity on the Northern Camino (58.32% of the municipalities), reflected in the number of skittles alleys (*n* = 291), the number of clubs, associations, and *peñas* participating in federated leagues (*n* = 162), and the wide range of varieties of skittles currently active on the Camino (*n* = 20). The relationship between skittles and local culture, both symbols of identity in these northern Spanish regions, made it possible to preserve the traditional heritage in these places, keeping it alive today, despite the push toward new and more attractive leisure and sport trends.

## Introduction

The interest in discovering diverse symbolic manifestations of the population, such as play, traditions, and folklore, has contributed to research locating these aspects—in addition to culture, community, society, and signifiers of identity—within the main approach to understanding a phenomenon that has, over time, been of particular interest in research about human beings’ behavior: the relationship between play and culture (Caillois, 1957; Bantulá, 2005, 2006; Enriz, 2011).

Disciplines such as social anthropology describe play as the result of social practices leading to activities of a more playful nature, adapted to people’s developmental stages (Schwartzman, 2012; Karpatschof, 2013; Hamayon, 2016). The play-culture binomial, noted by Vigne (2011) as a basic relationship refers to people’s nature, and also reflects the evolution of the human being from a play perspective (Salter, 1978; Parlebas, 2001; Lavega and Navarro, 2015; Rodríguez-Fernández, 2016) which is natural and adapted to reality. Play has clear cultural value (Stone, 1989; Huizinga, 2008); it is a symbol of culture (Enriz, 2011) and a kind of emblem, since through play and games we can come to understand the way of being of a given society (Parlebas, 2005; Sutton-Smith, 2009).

Culture is transmissible, transformable, and transformative. It is a guide, and it is senses, meanings, values, and practices (Álvarez; Enriz, 2011). In this regard, sociocultural processes such as enculturation, acculturation, and syncretism highlighted by Rodríguez-Fernández et al. (2014a), are reflected through playful practices. They help us to understand not only how people lived throughout history but also the origin and acquisition of certain customs as a result of people’s social interaction.

Coêlho de Araújo et al. (2013) refer to play as one of the most common expressions of human beings, an essential part of human nature which has been present in our culture since the oldest societies. From the wide range of play that exists, popular and traditional games best define the relationship between society and culture (Lavega, 1996; Rodríguez-Fernández, 2013). This is because they are a determining factor in individuals’ socialization and learning (Bantulá and Mora, 2002; Lavega, 2013) and because they allow us to make an intergenerational connection via the transmission of customs over time (Bantulá, 2006; Rodríguez-Fernández, 2013). Rodríguez-Fernández et al. (2014a) stated that traditions that are representative of regions or places with their own cultural character are transmitted principally by being a reflection of those places’ cultural idiosyncrasies. Habits, customs, and traditions are reinforced by routines, by everyday activities reflecting how people act, most commonly transmitted within the family, in which case it is from grandparents to parents and then to children (p. 1401).

Due to its relationship with culture, play may be considered one of a society’s intangible assets (Do Nascimento et al., 2015). In 2010, UNESCO included popular and traditional play in the list of world cultural heritage practices (Alonso et al., 2020). Bantulá (2006) associates that directly with caring about the study, preservation, promotion, and transmission of manifestations of play in the 21st century, and more generally by local authorities’ interest in the study of popular and traditional games in villages, regions and cities.

Out of the wide range of popular and traditional games we have access to, there is one that stands out due to its singular nature (Lavega, 1996) which has been widely played in many cultures and civilizations throughout history and is possibly one of the most widely played games in the world in its various forms (Ruiz, 2000). This is skittles, a traditional game based on throwing a ball at targets (skittles) in order to knock them down (knock-down skittles modalities) or move them as far as possible (*pasabolo* modality) (Rodríguez-Fernández et al., 2014a), which requires high levels of strength and precision, depending on the modality (Alonso-Rueda et al., 2015).

Despite the significant history of skittles, it has been in decline over recent years, making way for so-called modern sports and institutionalized games (Lavega, 2000; Parlebas, 2003; Zorrilla-López, 2005; Torre, 2013). This, along with the changing habits of a modern society that values the new and relegates traditions and the past to second place (Méndez-Giménez and Fernández-Río, 2011) has meant that skittles retains only a fraction of the prominent role it once had (Rodríguez-Fernández et al., 2014a).

There has also been a gradual decline in skittles being played, a trend which has been seen since the middle of the 20th century, which was a consequence of the loss of players and the closure of skittles alleys (Ruiz, 2000; Rodríguez-Fernández, 2013; Homobono, 2015). However, skittles is still played in many municipalities due to the efforts of various skittles clubs that particularly focus their activity on federated sports (mainly from the last third of the 20th century onward) (Braun, 1984).

In Spain nowadays, skittles is played more commonly in the northwestern part of the Iberian Peninsula, with most activity in Aragon, the Basque Country, Cantabria, Asturias, León and Galicia (Ruiz, 2000). The people who live in these areas, sensitive to the traditions that forged their identity, fought in order to help preserve their ancestors’ traditions, maintaining skittles as part of their day to day recreational activity (Braun, 1984; Zorrilla-López, 2005; Lucas-Recio, 2008; Rodríguez-Fernández et al., 2014b; Homobono, 2015; Rodríguez-Fernández, 2016).

In addition to people’s feelings about tradition in these areas, another aspect that helped to preserve skittles is its transformation from a game to a sport. It developed from a nonformal game, with flexible rules and a strongly social nature, to a formal, institutionalized activity with certain (rigid) rules, controlled and directed by a sports federation (Lavega, 2010; Sedano-Campo, 2010; Méndez-Giménez and Fernández-Río, 2011; Rodríguez-Fernández et al., 2015a). Nevertheless, authors such as Zorrilla-López (2005) have pointed out that this transformation also contributed to the loss of different varieties of skittles, especially in urban areas. Variants of skittles remain in the most rural areas, and are played only in specific tournaments during local festivals.

Expósito (2006), Moreno (2008), and Rodríguez-Fernández et al. (2015b) indicated a characteristic aspect of popular and traditional games, which is the use of open rural spaces, natural surfaces (earth, clay, sand, grass…), and the use of urban spaces which are not specifically designated for sport. This type of alternative space is commonly used for the skittles alley or *boleras*, generally in social meeting points such as squares, churches, or pubs. In the past, rural pubs were authentic sociocultural centers, where people could meet, interact, and share their hobbies, such as skittles (Ruiz, 2000; Rodríguez-Fernández et al., 2015b; Gonçalves-Junior et al., 2018). These locations combined commercial and recreational goals, providing an influx of people for the business, and contributing to the preservation of the most widely played traditional game in the world (Ruiz, 2000). Nowadays, with the transformation of skittles into a sport, many *boleras* have changed their appearance to more rigid, formal spaces, built using modern materials (both the space and the game material), and including places for spectators. However, they remain essentially places of meeting, socialization, and intergenerational interaction (Homobono, 2015).

Skittles has always been an exclusively male activity. Not because of the need to be strong to play certain types of skittles, but rather because of women’s roles in the past (Braun, 1998; Rodríguez-Fernández et al., 2014b, 2016; Gonçalves-Junior et al., 2018). The presence of women in skittles has changed radically. This has been due to both the continuous struggle against gender inequality and discrimination between men and women in sports (which also occurs in the skittles world), and by the recognition and appreciation of women who have kept certain varieties of skittles alive in an exclusively female way (De La Villa, 2002; Maestro, 2002, 2007; Martín, 2002a,b; Ungidos, 2002; Lucas-Recio, 2008).

Nowadays, skittles is still played in specific parts of Spain. Like most popular and traditional games, skittles has experienced a decline in parallel to the rise and consolidation of mass sports. Its survival rests mainly on maintaining its practice where it is still active (Ruiz, 2000; Rodríguez-Fernández et al., 2014b; Rodríguez-Fernández, 2016).

The Camino de Santiago (The Way of St James) is a religious and cultural movement that has been recorded in Spain since medieval times (Rodríguez-Fernández et al., 2014a). In the wider sense of the word, it was the label given to a series of Christian pilgrimage routes that went from all over Europe to the Cathedral of Santiago de Compostela to venerate the relics of St James the Apostle. The Camino de Santiago is an authentic source of spiritual, religious, and cultural exaltation with diverse beliefs and traditions, resulting from the footsteps of countless pilgrims from all over the world (Lester, 2015).

**FIGURE 1:**
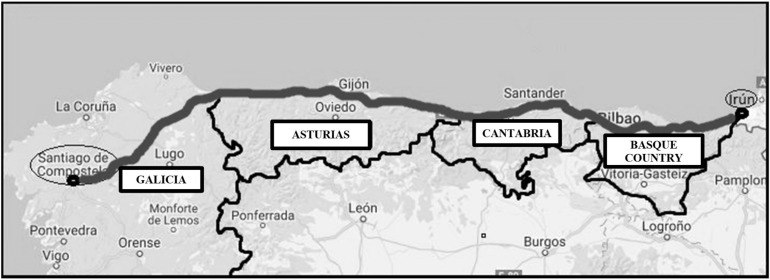
Northern route of the Camino de Santiago as it passes through the autonomous communities of the Basque Country, Cantabria, Asturias, and Galicia.

The Camino de Santiago is the longest of all the pilgrims’ routes. It was possible to trace this in the political and geographical framework of the medieval West. It began in the early 9th century and was strongly promoted from the 11th century (Acuña-Delgado, 2015). The route extended throughout Western Europe and was described early on in the Codex Calixtinus, preserved today in the Archive of the Cathedral of Santiago (Barreiro-Rivas, 2002). The Spanish Federation of Associations of friends of the Camino de Santiago (2020) recognizes 11 routes in the Iberian Peninsula and from all over Europe. These include the Northern Ways; the Camino Primitivo (the original way of St James), and the Northern Way (see Figure 1), considered one of the two most important routes along with the French Way (Fernández-Poyatos et al., 2011).

The French Way is a 750-km route linking Europe with northern Spain via the village of Roncesvalles. It has always been the main route of entry into the peninsula, nevertheless, since the 14th century, the municipality of Irún joined Roncesvalles as one of the main gateways through which pilgrims came from Europe, heading to Santiago de Compostela by several routes near the North coast of the peninsula, mainly the Primitive Way and the Northern Way. The difference between these two is that the Primitive Way is a mainly mountainous route from the Principality of Asturias, whereas the Northern Way continues along the entire Cantabrian coast (Pombo, 2018). The 817-km Northern Way from Irún to Santiago de Compostela is the longest of all the routes to the Galician capital (Whitson and Perazzoli, 2015), crossing four autonomous communities^1^ (the Basque Country, Cantabria, Asturias, and Galicia) and a total of 89 municipalities (29 in the Basque Country, 25 in Cantabria, 21 in Asturias, and 14 in Galicia).

Although the Camino de Santiago began with an exclusively religious purpose which continued for a long time, nowadays, many people prioritize the experience and enjoyment of the Camino over the religious objective (Acuña-Delgado, 2015), which highlights its polysemic character (Lois and López, 2012). From the walkers’ perspective, the route to Santiago is the “only first-magnitude historical pilgrimage whose itinerary is as important (or more important, as the case may be) as the very goal toward which it is heading” (Atienza, 2004, p. 12). In other types of pilgrimage (Christian or Muslim), the goal has always been the essential part of the journey, usually by the shortest route. The Camino de Santiago, in contrast, attaches special importance to the Way itself, which makes it a unique experience, since the spiritual goal sought by the pilgrim was not limited to reaching the end of the journey.

When talking about the Camino de Santiago, Martín-Luque (2014) noted that all the references and data were only collected from people who arrived in the Galician capital, minimizing the characteristics and history of those people who do not complete the route, or do it in reverse—back to their place of origin. Rodríguez-Fernández et al. (2014a) point to the formation of important population centers as a result of the pilgrimages to Compostela via the Camino de Santiago, in terms of infrastructure and human capital, something which led to the assimilation of foreign customs and which, according to Braun (1984), nowadays constitutes a huge cultural and artistic legacy.

In 1987, the Camino de Santiago was declared the “First European Cultural Itinerary” by the European Council, and in 1993, a “World Heritage Site” by UNESCO (Godoy-Portugal and Crecente-Maseda, 2018). This relationship between culture and the Camino de Santiago has not gone unnoticed by authors such as Granero-Gallegos (2004) and Ramos (2011), who consider the Camino to be the primary, most important European cultural route for its religious, historical, literary, and artistic aspects, which have greatly influenced Spanish culture through artistic and cultural currents produced by personal relationships, the exchange of ideas, and coexistence. Granero-Gallegos et al. (2005) emphasize that the Camino de Santiago constitutes an excellent framework for interculturality and coexistence that many young people and adults go to in search of personal experiences, fun, adventure, and to enjoy a physical activity in contact with nature.

In addition to the above, we can see how those cultural exchanges between the Iberian Peninsula and the rest of Europe through the Camino de Santiago have also had repercussions on sports, games, and recreational activities on the Camino (Brunhs, 2002; Lagardera, 2002; Rebollo, 2003; Granero-Gallegos, 2004; Granero-Gallegos et al., 2005; Jiménez-Vaquerizo, 2013). Braun (1984, 2003), Rodríguez-Fernández (2016); Rodríguez-Fernández et al. (2014a), Rodríguez-Fernández and Oliveira-Pereira (2016); Rodríguez-Fernández et al. (2018), Ruiz (2000); Sedano-Campo (2010), or Torre (2013), among others, have linked the origin of the traditional game of skittles to the Camino de Santiago, with significant German and French influences.

Studies by García-Serrano (1974) and Rodríguez-Fernández (2013, 2016) link the beginning of the game of skittles on the Iberian Peninsula to foreigners who arrived for some reason and brought with them a game that was not native to Spain but was played in other European countries. In this sense, the fact that the route of the Camino de Santiago facilitated the development of cultural practices, providing enrichment to the pilgrim routes, indicates that the game of skittles was one of those practices.

In research about skittles on the final stages of the different Caminos de Santiago, Rodríguez-Fernández et al. (2014a), recorded significant skittles activity mainly through four routes into Galicia: the Northern Way at its entrance through the region of Ribadeo in Lugo; the Portuguese Way through Tui in Pontevedra; and the area between the French Way and The Silver Way (Camino de la Plata), entering through Viana do Bolo, A Veiga, Carballeda de Valdeorras, Rubiá, O Barco de Valdeorras, Vilamartín de Valdeorras in Ourense, and Quiroga in Lugo. The study also stressed the presence of numerous federated sports clubs with formal sports activities (regulated competitions) linking that to the continuity and vitality of skittles in Galicia.

Subsequently, in a study about skittles in the final stage of the Portuguese Way (From Porto to Valença do Minho), Rodríguez-Fernández et al. (2018) described the practical nonexistence of skittles practice in this part of the Camino, finding it in only one of the nine municipalities on this part of the route and highlighting the practice of other traditional games with more roots in Portugal. They also stressed that skittles activity on the Portuguese Way in its entry into Galicia did not continue into Portugal.

The main purpose of this study is to analyze the game of skittles on the northern route of the Camino de Santiago, looking at its different varieties, the places it is played, and whether it is played as an informal or as a formal game. We also compare it with other traditional games, looking for links that help us reach a deeper understanding of the nature and essence of the game of skittles.

## Materials and Methods

### Design

We designed a mixed qualitative-quantitative study (Johnson and Onwuegbuzie, 2004; Pereira, 2011), with a descriptive, interpretative character (Lima and Palhares, 2014; Baptista, 2016). The qualitative analysis focused on the description of the different varieties of skittles over the entire pilgrimage route, those who promote it, where it is played, and the development of the game. The objective of the quantitative analysis was to compare variables and establish relationships in terms of variables including autonomous communities, modalities, transformation into sport, and the preservation of the traditional game, among others.

### Participants

We used intentional (nonprobabilistic) sampling to select informants from municipalities that the Northern Way runs through.

A total of 89 people participated in the study (73 men and 16 women, with a mean age of 48.67 and 42.06 years, respectively), one informant for each of the municipalities traversed by the Northern Way of the Camino de Santiago. The older age group was represented by subjects aged 50 or over (40.5%). The informants were mostly local authority professionals responsible for sport or culture (50.6% of the sample); failing that, the information from the municipal authority was obtained through a politician (councilor) from the sports or culture area (16.9%); 28.1% were players or managers of skittles clubs, with direct links to this traditional game. Minority of the samples (4.4%) were Physical Education teachers from local schools; this was in those municipalities where there was no skittles activity or, where due to size, there were no technical personnel for sports and culture or a delegate councilor. Due to the number of municipalities that the Northern Way crosses, the Autonomous Communities of the Basque Country (32.6%) and Cantabria (28.1%) were the largest contributors to the study (see Table 1).

**TABLE 1:** Participants’ sociodemographic characteristics.

	***N* (%)**
**Gender**	
Men	73 (82%)
Women	16 (18%)
**Age**	
Under 40	26 (29.2%)
Between 40 and 50	27 (30.3%)
Over 50	36 (40.5%)
**Profession**	
Municipal sports/culture professional	45 (50.6%)
Politician (Councilor for Culture or Sports)	15 (16.9%)
Player/club manager	25 (28.1%)
Other	4 (4.4%)
**Autonomous community**	
Basque Country	29 (32.6%)
Cantabria	25 (28.1%)
Asturias	21 (23.6%)
Galicia	14 (15.7%)
**Municipality’s population sizes by autonomous community**	
Basque Country	916.410
Cantabria	337.178
Asturias	487.617
Galicia	158.940

In addition, we selected informants using the same sampling approach from 60 municipalities from the same Autonomous Communities which had similar population characteristics but were not on the Northern Way. These respondents had a similar profile of age, sex, and profession to the Northern Way respondents.

### Measures

As there were no existing tools which met our requirements for this study, we designed a questionnaire *ad hoc* (Baptista, 2016; Magalhães and Hill, 2016).

The design of this questionnaire followed three phases: firstly, we identified the main elements in the existing literature on the subject; we analyzed and collated inconsistencies or contradictions present in those diverse theories, and finally we arranged the points into general categories creating specific information sections.

The resulting instrument collected information in four areas:

• Personal information (gender, age, type of informant, and municipality).
• Information related to skittles (whether the game is played currently, varieties of skittles, associationism, skittles alleys, formal and informal activity, and the importance of skittles in the municipality).
• Information about other traditional games and sports (varieties and how much they are played).
• Personal opinion about skittles and other popular and traditional games and sports (Knowledge, whether they play, preference, stage of development in the municipality, and transmission channels of the traditional game).

The sections were made up of different types of questions including open-ended, dichotomous, multiple-choice, and scalar-type questions. In order to verify reliability, the questionnaire was subjected to external validation by four expert judges in the academic context who were specialists in physical education and traditional games.

### Procedure

We contacted each municipality crossed by the northern route of the Camino de Santiago to establish contact with the person responsible for sport in the local authority. Where there was no specific person in the municipality responsible for sport, we used the councilor for sport and culture (political office) as a second option. In small municipalities, we also sought the participation of cultural representatives and delegate councilors.

In specific cases where the game was well established, the local authority contacts directed us to a person in charge of the local skittles club or sports club, or even regular skittles players, as they were able to provide us with better quality information.

We informed our contacts of the study objectives, that their participation was entirely voluntary, and that all personal data would be confidential and anonymous. Each participant gave their informed consent by marking a check box before completing the questionnaire.

We sent the questionnaire to the participants by e-mail and subsequently contacted them by telephone to thank them for their participation and collect additional data in the field notebook.

### Statistical Analysis

For the qualitative section of the study, three expert judges used content analysis. In the quantitative study, the responses to the questionnaire were coded and the data analyzed using the IBM-SPSS statistical package (version 25). We performed descriptive comparative analysis using the Mann–Whitney *U* test, Kruskal–Wallis, and Chi-square, along with relational analysis in order to compare responses between independent samples (gender, age, profession, and autonomous community).

Since the data did not fulfill the assumption of normality, we calculated the effect size for the Mann–Whitney *U* (*r* = *Z*/√*N*) and Kruskal–Wallis [ES = *H* ^∗^ (*N* + 1)/(*N* ^ 2−1)] tests for all significant results (Tomczak and Tomczak, 2014), interpreting them according to Cohen’s classification: 0.1, small; 0.3, medium; and 0.5, large. We performed pairwise comparisons of the Kruskal–Wallis test using the Dunn test with Bonferroni correction. Comparative test results are reported in the main body of the article only when *p* < 0.05.

## Results

### Current Situation of the Game of Skittles

Skittles is currently played in 52 (58.43%) of the 89 municipalities that lie on the Northern Way (see Table 2). Cantabria stood out as skittles is played in 24 (27%) of the 25 Cantabrian municipalities the pilgrimage route passes through. Galicia had the least skittles activity, since it is only played in 3 (3.37%) of the 11 municipalities on the Camino. In Asturias and the Basque Country, it is played in 40–60% of municipalities on the Camino. Although the Basque Country has the most municipalities on the Camino, and the highest population in these municipalities (see Table 1), there was a moderate amount of skittles activity. Cantabria, on the other hand, has a smaller population than Asturias or the Basque Country but reported more skittles activity. This shows that the skittles activities in the municipalities of the four communities the Northern Camino passes through are not directly proportional to their population sizes. In this regard, looking at the number of skittles clubs by the population of the municipalities, there are 2.9 skittles clubs per 100,000 inhabitants in the municipalities in the Basque Country, but 33.8 skittles clubs per 100,000 inhabitants in the municipalities in Cantabria. The corresponding figures for Asturias and Galicia are 8.8 and 1.9, respectively. Similarly, applying the same approach to the number of skittles alleys, in the Basque Country, there are 3.6 bowling alleys per 100,000 inhabitants, while in Cantabria, there are 52.5, in Asturias 13.7, and in Galicia 8.8.

**TABLE 2:** Skittles activity by autonomous community.

		**Skittles currently played**		**Total**
		**Yes**	**No**	
Autonomous community	Basque Country	13	16	29
	Cantabria	24	1	25
	Asturias	12	9	21
	Galicia	3	11	14
**Total**		**52**	**37**	**89**

In terms of being played in the past, 64 informants (71.91%) reported that skittles used to be played (that they have evidence of) in their municipalities. We found 20 different types of skittles played along the Northern Camino (see Table 3). In the Basque Country, there were 10 different modalities (without a clear predominant variety), with the greatest activity recorded in the last third of the Camino passing through this community. In Cantabria, we recorded only four skittles varieties, the most common by far being *Bolo Palma* (in 88% of the municipalities on the Camino), followed by *Pasabolo Losa* and *Pasabolo Tablón*. In the last third of the Cantabrian Camino, only *Bolo Palma* was played. In the Principality of Asturias, we recorded seven types of skittles. The most widely played was *Cuatreada* (in 8 of the 21 municipalities on the Camino, representing 38.09%). In Asturias, most skittles activity was concentrated in the first two-thirds of the Camino (with a preference for *Cuatreada* and *Batiente*) and in the last third we recorded very little activity (only in one of the last seven municipalities on the Camino in this part of Asturias). In Galicia, we recorded only three types of skittles (*Bolo Celta*, *en Línea*, and *Ó Canteiro*) in the first three municipalities at the beginning of the route in Galicia. From there to Santiago de Compostela, in the remaining 11 municipalities, we found no record of skittles being played nowadays.

**TABLE 3:** Active skittles varieties by autonomous community.

**Variety**	**Basque Country (No. municipalities)**	**Cantabria (No. municipalities)**	**Asturias (No. municipalities)**	**Galicia (No. municipalities)**	**Total**
Bolatoki	2	0	0	0	2
Hiru txirlo	3	0	0	0	3
Toka	3	0	0	0	3
Bolos a katxete	3	0	0	0	3
Remonte	1	0	0	0	1
Tres tablones	4	0	0	0	4
Calva	2	0	0	0	2
Bolo leonés	2	0	1	0	3
Pasabolo tablón	3	4	0	0	7
Bolo palma	2	22	2	0	26
Pasabolo losa	0	6	0	0	6
Bolo pasiego	0	1	0	0	1
Cuatreada	0	0	8	0	8
Bolo Tineo	0	0	1	0	1
Batiente	0	0	4	0	4
Bolinos	0	0	1	0	1
Rodao	0	0	1	0	1
Bolos celtas	0	0	0	1	1
Bolos á línea	0	0	0	1	1
Bolos ó canteiro	0	0	0	2	2
**Total**	**25**	**33**	**18**	**4**	**80**
					

We found a total of 162 clubs, associations or *peñas* participating in federated skittles competitions and 25 participating in nonformal (nonfederated) competitions (see Table 4). These organizations were located in 48 of the municipalities on the Camino (53.93%), and practically all of them (47) engaged in competitive (federated) activity. We found only one municipality with skittles clubs that did not participate in federated activity. There are regular tournaments in the municipalities where these clubs were recorded (many coinciding with important dates on the calendar, such as the popular festivals in each place) in addition to federated competition. To a lesser extent, these clubs also participate in internal (nonformal) leagues that run parallel to formal (federated) competition.

**TABLE 4:** Skittles clubs and participation in formal and nonformal competitions.

	**Mun. with skittles clubs^a^**	**Mun. with skittles clubs fed.^b^**	**Mun. without skittles clubs fed.^c^**	**No. skittles clubs fed.^d^**	**No. skittles clubs nonfed.^e^**	**Mun. tournaments^f^**	**Mun. leagues int.^g^**	**Mun. leagues fed.^h^**
Basque Country	11	10	1	26	1	9	9	10
	12.36%	11.24%	1.12%	16.05%	4%	10.11%	10.11%	11.24%
Cantabria	24	24	4	92	22	24	16	23
	26.97%	26.97%	4.49%	56.79%	88%	26.97%	17.98%	25.84%
Asturias	11	11	2	41	2	11	8	11
	12.36%	12.36%	2.25%	25.31%	8%	12.36%	8.99%	12.36%
Galicia	2	2	0	3	0	2	0	2
	2.25%	2.25%	–	1.85%	–	2.25%	–	2.25%
Total	**48**	**47**	**7**	**162**	**25**	**46**	**33**	**47**
	**53.93%**	**52.81%**	**7.86%**	**–**	**–**	**51.68%**	**37.98%**	**52.81%**

*^*a*^Number of municipalities with clubs dedicated exclusively skittles. ^*b*^Number of municipalities with skittles clubs participating in formal (federated) competitions. ^*c*^Number of municipalities with skittles clubs participating in nonformal (friendly) competitions. ^*d*^Number of skittles clubs competing in formal (federated) competitions. ^*e*^Number of skittles clubs competing in nonformal (friendly) competitions. ^*f*^Number of municipalities where skittles tournaments are held regularly. ^*g*^Number of municipalities with internal skittles leagues (nonfederated). ^*h*^Number of municipalities where skittles league days are held (federated).*

Examining the data in Table 4 by autonomous community, 51% of the municipalities with federated skittles clubs were in Cantabria (*n* = 47), 21.3% were in the Basque Country, and 23.4% were in Asturias. Only 4.3% of municipalities with federated skittles clubs were in Galicia. These data are proportional to the number of federated clubs by autonomous community; there were 92 in Cantabria, 26 in the Basque Country, 41 in Asturias (in this case there was a higher density of federated clubs by municipality compared with the Basque Country), and 3 in Galicia. The proportions of nonfederated clubs were similar to the federated clubs, albeit with much smaller numbers: 22 in Cantabria, 2 in Asturias, 1 in the Basque Country, and none in Galicia.

In the 89 municipalities analyzed, we recorded a total of 291 skittles alleys dedicated exclusively to the game (see Table 5). Most (60.82%) were in Cantabria, 23.02% in Asturias, 11.34% in the Basque Country, and 4.81% in Galicia. Skittles alleys were reported in 57 of the 89 municipalities on the Camino (64.04%). In Cantabria, 24 out of the 25 municipalities had skittles alleys (with a ratio of more than 7 fields per municipality), in Asturias 15 out of 21, in the Basque Country 13 of 29 and in Galicia, 5 out of 14, including municipalities with skittles alleys but where skittles is not currently played (evidence of past activity).

**TABLE 5:** Skittles alleys.

	**No. of municipalities with skittles alleys**	**No. of municipalities without skittles alleys**	**Total no. of skittles alleys**
Basque Country	13	16	33
	14.61%	17.98%	11.34%
Cantabria	24	1	177
	26.97%	1.12%	60.82%
Asturias	15	6	67
	16.85%	6.74%	23.02%
Galicia	5	9	14
	5.62%	10.11%	4.81%
Total	**57**	**32**	**291**
	**64.04%**	**35.96%**	**–**

Table 6 shows the informants’ perceptions of the importance attached to skittles in each place (on a scale of 1 to 5). The scores were low (and directly proportional to the activity in each autonomous community), with an average of 3.72 in Cantabria, a medium-high assessment. Asturians had a low-medium score (2.24), those in the Basque Country had a low score (2.03), and Galicians had a very low score (1.57).

**TABLE 6:** Importance of the game of skittles in the municipality.

	**Average rating (1–5)**	**SD**
Basque Country	2.03	1.24
Cantabria	3.72	1.02
Asturias	2.24	1.41
Galicia	1.57	1.09
Total	**2.48**	**1.43**

### Other Popular and Traditional Games and Sports

In addition to skittles, informants reported other popular and traditional games and sports on the route of the Northern Camino (see Table 7). We classified the informants’ responses into four types: *Pelota-pala-cesta* (ball games using hands, paddles, shovels, or baskets); *Rural Sport* (which includes activities such as rope pulling, log cutting with saws or axes, races with milk jugs, lifting stones or sacks of corn, stone dragging (whether by people or oxen), mowing grass, and stick pulling, etc.); *Rowing* (in fixed seats in boats called *Traineras*); and *Others* (activities that do not fit into the previous sections and that constitute practices inherited from generation to generation, such as the key game, the frog, *tuta*, *toka*, and *petanca*, among others).

**TABLE 7:** Other popular and traditional games and sports on the Northern route of the Camino.

	**Ball games**	**Rural sport**	**Rowing**	**Others**
Basque Country	23	15	10	5
	79.31%	51.72%	34.49%	17.24%
Cantabria	0	2	7	3
	–	8%	28%	12%
Asturias	0	4	2	6
	–	19.05%	9.52%	28.57%
Galicia	0	0	0	4
	–	–	–	28,57%
Total	**23**	**21**	**19**	**18**
	**25.84%**	**23.60%**	**21.35%**	**20.22%**

In the Basque Country, *Pelota* (ball games) stood out (in 79.31% of the municipalities on the Camino); these were exclusive to the Basque Country and not reported in any of the other autonomous communities. We also saw notable levels of rural sports (51.72%) and rowing (34.49%). This is not surprising, since the Northern route of the Camino (also called the *Coastal Way*) runs along the entire coast of the Cantabrian sea, hence the keenness for this nautical sport, originally with rowing boats used for fishing and evolving into a sport over time.

In Cantabria, rowing stood out (28% of the municipalities on the Camino) and, to a lesser extent, rural sport (8%) and other popular and traditional games and sports (12%). In Asturias, the highest percentage was in other games (28.57%), with fewer reports of rural sports (19.05%) and rowing (9.52%). In Galicia, there was only activity reported in other games and pastimes (28.57%), referring to traditional games such as *billarda*, the key game, badges, the frog, and *peonza*, among others.

In terms of which traditional/popular game or sport the respondents considered to be most important in their municipalities (see Table 8), skittles stood out in the entire Northern Camino (40.45%). By autonomous community, in the Basque Country, ball games stood out (42.27%), in Cantabria and Asturias, skittles was the most important (84 and 52.38%, respectively), and in Galicia, other popular and traditional games and sports as noted above were more important, particularly *billarda*.

**TABLE 8:** Importance of popular and traditional games and sports on the Camino.

	**Skittles**	**Ball games**	**Rural sport**	**Rowing**	**Others**	**None**
Basque Country	4	14	3	7	1	0
	13.79%	48.27%	10.34%	24.14%	3.45%	–
Cantabria	21	0	0	3	0	1
	84%	–	–	12%	–	4%
Asturias	11	0	0	2	1	7
	52.38%	–	–	9.52%	4.76%	33.33%
Galicia	0	0	0	0	8	6
	–	–	–	–	57.14%	42.86%
Total	**36**	**14**	**3**	**12**	**10**	**14**
	**40.45%**	**15.73%**	**3.37%**	**13.48%**	**11.23%**	**15.73%**

### Transmission Channels for Popular and Traditional Games and Sports

Our data, which came from a multiple choice item, indicates that popular and traditional sports and games were mainly passed on through the family (69.66%). Other means of transmission such as the media or personal practice accounted for 65.17% of the responses. Family and personal experience of playing the game were presented as the best tools for the transmission of traditions from generation to generation. On the other hand, school as a transmission channel generated 35.95% of the responses while community transmission (friends, neighbors, etc.) appeared in 30.34% of responses.

### Skittles Based on Sociodemographic Variables

In order to identify how gender, age, and profession influence knowledge, practice, and preference for skittles, we performed a comparative analysis using the Mann–Whitney *U* test.

Men scored higher than women (see Table 9) in terms of knowledge of skittles (*U* = 399.500, *p* = 0.035, *r* = 0.22), playing it (*U* = 288.000, *p* = 0.001, *r* = 0.35), and their preference for the game (*U* = 281.500, *p* = 0.001, *r* = 0.36).

**TABLE 9:** Results of Mann–Whitney *U* test comparing gender and skittles.

	***U* of Mann–Whitney**	***Z***	**Asymptotic significance (bilateral)**	***N***	***r***
Knowledge	399.500	−2.11	0.035	89	−0.22
Preference	288.000	−3.32	0.001	89	−0.35
Practice	281.500	−3.36	0.001	89	−0.36

The only significant differences we found with respect to age were about knowledge of skittles [*χ*^2^(2, *N* = 89) = 6.947; *p* = 0.031]. This indicates that older people knew more about it (see Table 10).

**TABLE 10:** Results of Kruskal–Wallis comparing age and skittles.

	***χ*^2^**	**Gl**	***N***	**Asymptotic significance (bilateral)**	**$E_{\text{R}}^{2}$**
Knowledge of skittles	6.947	2	89	0.031	0.08

We found significant differences between respondents based on profession (see Table 11) in terms of playing skittles [*χ*^2^(3, *N* = 89) = 28.652; *p* < 0.001]. Specifically, we found a significantly higher score in this regard (*p* < 0.001) for club players/managers (67.18) compared with sports/cultural officers in the municipality (35.79) or to councilors for culture or sports (37.60).

**TABLE 11:** Results of Kruskal–Wallis comparing profession and skittles.

	**Knowledge**	**Practice**	**Preference**	**Played in municipality**
*H* of Kruskal–Wallis	24.965	42.930	31.244	28.652
Gl	3	3	3	3
Asymptotic sig	0.000	0.000	0.000	0.000
$E_{\text{R}}^{2}$	0.28	0.49	0.36	0.33

### Relationship Between Playing Skittles and Other Sports

Table 12 presents the analysis of differences between those who played skittles and those who did not with regard to knowledge of other traditional games (*U* = 545.50, *p* = 0.000, *r* = −0.42), whether they played traditional games in the past (*U* = 366.500, *p* = 0.000, *r* = −0.54), their preference for these games (*U* = 436.000, *p* = 0.000, *r* = −0.50), and what they usually play (*U* = 547.000, *p* = 0.000, *r* = −0.39). We found that subjects who played skittles had greater knowledge of other traditional games, played them to a greater extent in the past, showed more interest in them, and currently play this type of sport or game more often.

**TABLE 12:** Results of Mann–Whitney *U* test comparing other traditional sports/games and playing skittles.

	**Knowledge of traditional game**	**Played traditional game in past**	**Liking traditional game**	**Plays traditional game now**
*U* of Mann–Whitney	545.500	366.500	436.000	547.000
*W* of Wilcoxon	1248.500	1069.500	1139.000	1250.000
*Z*	−3.992	−5.049	−4.681	−3.716
Asymptotic sig (bilateral)	0.000	0.000	0.000	0.000
*r*	−0.42	−0.54	−0.50	−0.39

### Skittles Activity and Its Relationship With the Northern Route of the Camino de Santiago

In order to analyze whether the northern route of the Camino de Santiago influenced the playing of skittles, we performed a comparative analysis between municipalities on the Camino (*n* = 89) and a sample of other, similar municipalities (*n* = 60) from the same autonomous communities.

Firstly, we found significant differences between the two groups in the number of clubs (see Table 13). The municipalities on the northern Camino route had more clubs compared with the other municipalities (*U* = 745.500, *p* = 0.000, *r* = −0.44).

**TABLE 13:** Results from Mann–Whitney *U* test comparing number of clubs in municipalities on the Northern Camino and other municipalities.

	***U* of Mann–Whitney**	***Z***	**Asymptotic significance (bilateral)**	***N***	***r***
No. of clubs	745.500	−4.615	0.001	109	−0.44

We also found differences in terms of the number of varieties of skittles [*χ*^2^(2, *N* = 149) = 10.253; *p* = 0.006]. In fact, the municipalities on the Camino had a greater variety of types of skittles, which we relate to the cultural diversity of the pilgrim route, which would have helped to preserve and develop this activity, allowing it to reach other areas of Spain.

In line with the above, our analysis also indicated significant differences pointing to greater wealth in terms of traditional games and sports played in the municipalities on the northern route [*χ*^2^(1, *N* = 146) = 10.786; *p* = 0.001]. This shows, on the one hand, the cultural influence on the areas the Camino runs through contributing to enriching the games in these areas, and on the other hand, the preservation of the traditions associated with the experience involved in this pilgrimage route.

## Discussion

One of the main results of this study shows that skittles is still played in almost 60% of the municipalities that make up the Northern Camino and, moreover, it is the most popular traditional game played on this route, ahead of ball games (which are exclusive to the Basque Country), rural sports, rowing, or the other types of traditional games and sports often played in this area. Skittles is one of the most deeply rooted traditional games in this region. As various studies have shown, skittles has served as an important social meeting point in different communities by functioning as a channel of cultural transmission, contributing to its promotion and conservation (Ruiz, 2000; Rodríguez-Fernández, 2013).

Skittles, as with other “popular” games, serves as a tool of identity for social groups. Playing it contributes to the maintenance of habits and cultural forms that are transmitted from generation to generation.

García-Serrano (1974) reported that Spain was rich in popular and traditional games and they are currently in good health (Doncel-Recas, 2014), with the Cantabrian coast having the largest number of these games, particularly the Basque Country and Navarra (Baena-Extremera, 2005). With respect to communities on the Northern Camino, Moreno (1993) placed the Basque Country at the highest level of activity followed by Cantabria, Asturias, and Galicia at a high level. Skittles has been included in these assessments, with most activity being reported from Cantabria, Asturias, and the Basque Country (Rodríguez-Fernández, 2013; Rodríguez-Fernández et al., 2015a). In the case of the Basque Country, different studies and reports have been published collecting evidence of the wide diversity of traditional games and sports played in that region, among which the game of skittles stands out (Mendizabal and Homobono, 1966; Aguirre, 1971; Ibabe and Lujanbio, 1987; Glaria, 1988; Peña et al., 2018).

Our study recorded skittles activity in the municipalities of Ribadeo, Barreiros and Lourenzá, the first three municipalities upon entering Galicia via the Northern Camino. In the fourth municipality (Mondoñedo), we found no reports of current skittles activity, although there were indications of recent activity considering that we did have a report of a skittles alley here. These data match the findings in study by Rodríguez-Fernández et al. (2014a) about skittles in the final stages of the Camino de Santiago, linking skittles with the municipalities that the Northern Camino goes through as it enters Galicia and other municipalities near this route.

In his study of skittles in Vizcaya (Basque Country), Zorrilla-López (2005) reported skittles activity in the municipalities on the Northern Camino in this community. However, the highest concentration of skittles was in the municipalities in the interior of the Basque Country. Our data agree with these findings, noting skittles activity near the coast at 44.83%, data that Zorrilla-López (2005) noted as worrying when referring to skittles as a type of game which used to be as popular as ball games, the main traditional game played in the Basque community which in our study was played in 48.27% of municipalities.

Skittles activity was reported in 96% of Cantabrian municipalities on the Northern Camino. This very high percentage confirms the levels of skittles activity in this community reported by authors such as Blanco-Maza (1992), Hoyos (2002), and Torre (2013) and indicated by the records of the activity (league matches, cup, regional and national championships, circuits, competitions, and tournaments) of the Cantabrian Skittles Federation (2020a). Braun (2019) pointed out that playing skittles in Cantabria is influenced by the love for the land of the people who play, it is a symbol of identity for this community, hence the Cantabrian government’s commitment to preserving this tradition, supporting the regional federation, and sports clubs, and promoting an educational project in schools to consolidate the future of these practices in the community (Zuloaga-Martínez, 2019).

In Asturias, 57.14% of municipalities on the Northern Camino reported skittles being played; these are, together with Cantabria, the highest rates in our study. Authors such as Ruiz (2000) and Mencía (2007) highlighted how much skittles was played in this community, while they also indicated concern about its gradual decline in recent years. Both authors highlighted how popular skittles was in general throughout the Principality of Asturias, both because of the federated activity each year and because of the relationship with the local culture, since skittles is a very old tradition in this region which belongs to all Asturians (Mencía, 2007). Plans to promote it were put in place in schools to maintain that tradition in addition to regulated competitions, especially through Physical Education lessons (Crespo, 2002; Mencía, 2007).

In the four communities on the Northern Camino, we recorded 20 different types of skittles. On the one hand, this indicates the plurality of the game in terms of its aspects and modalities, something highlighted by authors such as Lavega (1996), Ruiz (2000), Rodríguez-Fernández (2013, 2016), and Torre (2013), and for Blanco-Maza (1992), it remains one more identifying factor of the idiosyncrasies of each of these peoples. From the 10 modalities played in the Basque Country, 7 were only played in this region, particularly *Hiru txirlo*, *bolatoki*, *toka*, and *bolos a katxete* (Zorrilla-López, 2005), all of which are knock-down skittles games. We found four types of skittles in Cantabria, two *pasabolo* type (*pasabolo-losa* and *pasabolo-tablón*) and two knock-down type (*bolo palma* and *bolo pasiego*) (Cantabrian Skittles Federation, 2020b). *Bolo palma* is the most deeply rooted in Cantabria and one of the most widespread in Spain (De la Torre, 2002; Zorrilla-López, 2005). This agrees with our data, where *bolo palma* is played in 22 of the 25 Cantabrian municipalities on the Northern Camino (88%). In Asturias, we found seven different varieties of the game, the most common being *cuatreada*, which agrees with the studies by Mencía (2007) and Ruiz (2000). In Galicia, we recorded three types (*bolo celta*, *á línea* and *ó canteiro*, all *pasabolo* types) matching the findings from Rodríguez-Fernández et al. (2014a) in his study on skittles in the final stage of the Camino de Santiago.

A unique aspect of our study is our record of 162 clubs, associations, and *peñas* that participate in federated skittles competitions. The fact that the game is mediated by sports clubs that participate in regulated competitions (under the control of a sports federation) means that we are talking about a sport. In this sense, skittles is a sport, which abandons the essence of what a game represents in itself (a nonformal activity characterized by flexibility in the rules to adapt them to a given context). This transformation of a traditional game like skittles to a sport (Maestro, 2007; Lavega, 2010; Sedano-Campo, 2010; Méndez-Giménez and Fernández-Río, 2011; Rodríguez-Fernández et al., 2015a) contributed to the decline or practical disappearance of many indigenous varieties (Martín, 2002c; Zorrilla-López, 2005), but on the other hand, there was a definitive commitment to keeping the sports modalities alive (Ruiz, 2000; Hoyos, 2002; Mencía, 2007; Rodríguez-Fernández, 2013; Rodríguez-Fernández et al., 2015a). In this regard, Lavega (2002, p. 93) stresses the maintenance of the game-sport binomial, proposing it as an empowerment, promotion, and dissemination of the game of skittles “reconciling sport with celebration, tradition with innovation, sharing with fun competition.”

The activity in the municipalities of the Northern Camino was also reflected in the number of skittles alleys we recorded in this study, a total of 291. Almost two-thirds (64.04%) of the municipalities through which this pilgrimage route passes have one or more skittles alleys. These play spaces have always been considered social spaces (Ruiz, 2000; Rodríguez-Fernández, 2013; Rodríguez-Fernández et al., 2015b), defined by Torre (2013) as centers for leisure, meeting and social gatherings, fun, and sporting challenges, although some voices have emerged warning of a change in the concept of skittles alleys in which competition has won to the detriment of play, abandoning that nuance of “place of social relationship” which has always characterized skittles alleys. In any case, skittles alleys have improved in quality over time. Hoyos (2002), on the state of the *Boleras* (skittles alleys) in Cantabria, where 60.82%—according to our study—are located, indicated that in many cases they have gone from being a small stone wall with trees for shade to small covered stadiums, with advertising, management, and stands capable of accommodating three thousand people comfortably seated.

Despite the high levels of skittles activity we found on the Northern Camino, the participants in our study generally had low opinions of the importance of skittles in their corresponding localities, with the exception of Cantabria, which give a medium-high rating. These data coincide with the study by Rodríguez-Fernández (2013) on skittles in a Galician town, where participants had a low opinion of skittles, considering it a simple game, irrelevant to other facets of life except for specific leisure time. In relation to other games and sports played in the four autonomous communities covered by this study, our data confirms that skittles is the most popular traditional game/sport played in Cantabria and Asturias, the two communities that gave it a higher score. Mencía (2007) and Torre (2013) attributed the importance of skittles in these localities to how much the inhabitants and entities (public and private) cared about maintaining this type of tradition, and their awareness of skittles as a symbol of their identity and part of their culture and heritage. In the Basque Country, respondents had the highest opinions of traditional Basque ball games, also due to their close relationship with traditional Basque culture (González-Abrisketa, 2006, 2013).

Our analysis of skittles in terms of demographic variables indicated that men knew more about the game, played it more, and had higher levels of preferences for it than women. This is not surprising as it is a game which is mostly played by men (Braun, 1984; Blanco-Maza, 1992; Ruiz, 2000; Rodríguez-Fernández, 2013). Although women do play skittles (Braun, 1998; Ruiz, 2000; De La Villa, 2002; Maestro, 2002, 2007; Martín, 2002a,b; Ungidos, 2002), their participation is generally symbolic except for specific cases throughout Spain (Rodríguez-Fernández et al., 2016). The older our respondents were, the better their knowledge of skittles. People who played skittles also knew more about other popular traditional games and sports. In this regard, it is important to consider the decline in playing skittles and its loss of prominence (Lavega, 2000; Parlebas, 2003; Zorrilla-López, 2005; Torre, 2013; Rodríguez-Fernández et al., 2014a), which means that older people not only know more about skittles but also other traditional games and sports in general thanks to their regular contact with them. Similarly, in terms of participant profiles, players or managers of federated clubs played more. This confirms the idea of skittles being made into a sport, to the detriment of being considered a strictly traditional game.

Our results confirm a development of skittles in the towns and villages on the northern route of the Camino de Santiago which has led to it becoming a “sport.” However, there are few studies on this topic in this area, and those that do exist confirm that skittles is widely played in the Basque Country, Cantabria, and Asturias, but less so in Galicia, where it is mostly played symbolically. In that regard it is important to note that Galicia is the last stage of the northern route (Figure 1), something that may help to explain the low levels of skittles activity in the area. Nonetheless, this type of research not only helps us get closer to the reality of an important traditional game in the peninsula—particularly in the North of Spain—but also helps clearly illustrate part of the local culture associated with popular games.

## Conclusion

The Northern route of the Camino de Santiago is a route of interaction and cultural exchange not only between the Iberian Peninsula and the rest of Europe but also between the inhabitants of the different regions of northern Spain. The possible French and German influences on the game of skittles in other times have left us with current data indicating that skittles is widely played along this route (almost 60% of the municipalities on the Northern Way). This activity is reflected in the number of skittles alleys (291) and in the 162 clubs, associations and *peñas* that participate in leagues, championships, and competitions organized by the regional federations and by the Spanish Skittles Federation.

From the 20 different varieties of skittles we found along the Camino (with knock-down types being more common than *pasabolo* types), *bolo palma* stood out as the most widely played, especially in Cantabria, a community that placed the highest value on skittles and that reported more skittles played than the other Spanish regions on the Northern Camino (96% of Cantabrian municipalities on the Camino). In addition to interest in skittles, our research confirms the importance felt in the Basque Country, Cantabria, and Asturias about maintaining their traditions through traditional games and sports. In the Basque Country, it was through Basque ball games (handball, shovel or basket), rural sports, rowing and skittles. In Cantabria, it was through skittles, and in Asturias, through skittles and rural sports. In Galicia, there was also interest in skittles, although at lower levels, along with other games such as *billarda*, the key, and the frog.

Finally, our study shows how traditions are passed on from generation to generation, mainly through the family. Personal experiences and contact with popular and traditional games and sports are essential for parents in order to transmit to their children the full value they place on this type of activity. For this reason, public administrations must focus on the promotion, dissemination, and preservation of popular culture through traditional games, collaborating and supporting private nonprofit entities (federations, clubs, associations, and skittles clubs), and establishing mechanisms that motivate new generations to play traditional games and sports, with the school being an important place to focus this important task.

## Data Availability Statement

The raw data supporting the conclusions of this article will be made available by the authors, without undue reservation.

## Ethics Statement

This study was carried out in accordance with the recommendations of Bioethics Committee of the University of Santiago de Compostela (Spain). All subjects gave written informed consent in accordance with the Declaration of Helsinki.

## Author Contributions

JR-F and ML-M conceived and designed the research. JR-F carried out the data collection and management with informants. JG-Á and GM-S analyzed the data and reflecting it in the study results. JR-F drafted the manuscript. ML-M, JG-Á, and GM-S contributed to the writing of the study and reviewed it. All authors read and approved the manuscript.

## Conflict of Interest

The authors declare that the research was conducted in the absence of any commercial or financial relationships that could be construed as a potential conflict of interest.

## Footnotes

1 Spanish territorial administrative entity with certain legislative autonomy and its own elected representatives.
